# Application of 3D Printing Technology in Furniture Construction

**DOI:** 10.3390/ma17194848

**Published:** 2024-10-01

**Authors:** Boryana Petrova, Vassil Jivkov

**Affiliations:** 1 Department of Interior and Furniture Design, Faculty of Forest Industry, University of Forestry, 1797 Sofia, Bulgaria; b.petrova@ltu.bg; 2 Department of Interior and Architectural Design, Faculty of Architecture, University of Architecture, Civil-Engineering and Geodesy, 1046 Sofia, Bulgaria

**Keywords:** 3D printing, PLA, furniture, connecting elements, joint strength, bending moment, stiffness coefficient

## Abstract

In recent years, 3D printing technology has become very important in many fields of science, manufacturing, design, medicine, aviation, sports, etc. Furniture design and manufacturing are also not left out of this trend. In this study, the results of bending moments and stiffness of joints of thin structural elements connected by 3D printing with polylactic acid (PLA) connectors are given. The connectors are newly developed, and information on their strength characteristics is lacking in the literature. Ten joints were investigated, made with 9 and 12 mm plywood and 6 mm MDF. The tested joints constructed by 3D-printed connecting elements show a high strength under arm compression bending load, between 44.16 and 24.02 N·m. The stiffness coefficients of joints with 3D-printed connecting elements are between 348 and 145 N·m/rad and are higher than those of conventional detachable mitre joints but lower than those of glued ones. The type of filling of the hollow section of the connecting elements and the wall thickness influenced the joints’ strength and stiffness. Reducing the width of the connecting elements from 40 to 30 mm and the inner radius between the arms from 2 to 1 mm does not significantly affect the joints’ strength and stiffness coefficients.

## 1. Introduction

The development of 3D printing technology has especially become popular in recent years and has entered massively into various fields of science, manufacturing, design, medicine, aeronautics, sports, etc. Furniture is also keeping up with this trend, and many designers are experimenting with different shapes and functions [1,2,3,4,5,6]. Recently, new developments of joints obtained through 3D printing technology have also emerged and are of interest to the furniture industry [1,4,5,7,8,9,10,11,12,13,14,15].

### 1.1. 3D Printing: An Opportunity to Prototype and Experiment with Different Structural Joints

Design freedom, waste optimisation, the ability to rapidly prototype, perform specific tasks, reduce material costs, lighten construction, give high-strength printed structures, and produce complex and sophisticated structures are among the main advantages of 3D printing [16,17,18]. Three-dimensional printing technology, also known as additive manufacturing (AM), is based on overlaying layers of polymer, metal, wood, or ceramic to create a three-dimensional object from a computer model or from an object that has been scanned with a 3D scanner, or even a regular camera [19,20].

ISO/TC 261 and ASTM F42 standards create a structure that defines a hierarchy of AM standards with three levels [21]. The first level is Feedstock Materials, the second is Process/Equipment, and the third is Finished Parts. Materials are divided into the following categories: metal powders, ceramic powders, photopolymer resins, polymer powders, metal rods, polymer filters, etc. According to the process and equipment category, AM is divided into the following categories: material jetting, powder bed fusion, binder jetting, directed energy deposition, material extrusion, sheet lamination, and vat polymerisation. The finished parts are for mechanical test methods, non-destructive evaluation (NDE)/non-destructive testing (NDT) methods, post-processing methods, bio-compatible test methods, chemical test methods, etc.

Using additive technology, thermoplastic products with excellent mechanical, thermal, and chemical properties that are relatively inexpensive and do not require expensive machinery can be produced. The main methods of AM using 3D printers are overlaying extruded thermoplastics (Fused Deposition Modelling—FDM) and Selective Laser Sintering (SLS). In SLS, polyamide 12 (PA12) is mainly used as a material. Although there is an extreme variety of polymers on the market, PA12 remains one of the best powder polymers that meets the requirements for SLS production [22]. In 3D printing technologies, wood can be used as a raw material for printing [23].

Many studies and scientific publications have been carried out in AM. Some relate to this method’s definition of manufacturing objects’ main guidelines and problems [18,24,25,26]. Others investigate specific parametric models to optimise the shape while improving different fastener models’ physical and mechanical properties [27,28,29,30,31,32]. The influence of adhesive type on the strength of the final products has also been investigated [33]. Many of the authors have also carried out theoretical research using the finite element method (FEM), which attempts to optimise the shape of the objects in order to remove locations with stress concentrations and create preconditions for failure during operation [7,12,33,34,35,36].

Snap-fit fasteners, known for their many applications in various areas of life, such as seat belts in cars, child seats, strollers, and many others, can also be attractive for furniture design, especially when prototyping and experimenting. Making a snap-fit mechanism made of plastic that provides the necessary tightening force is relatively difficult. In contrast to joints where the tightening force is provided by threading, fastening mechanisms must rely on bending, but this is not a recommended method [24]. Even if they can provide the required tightening force at the beginning of their service life, they lose their elastic properties over time, leading to a loss of elasticity and, respectively, tightening force [24].

In recent years, there has also been an increased interest in 3D printing technology for prototyping and manufacturing joints for furniture. Polish scientists developed and patented a new joint obtained by 3D printing [11]. The joints were made from acrylonitrile-butadiene-styrene (ABS) on a Flashforge 3D Sygnis printer (Hangzhou, China). The housing dimensions were 4 or 30 mm long and 13.4 mm in height. Another team of Turkish researchers developed and produced the connecting elements by 3D printing and optimised their shape and mass using specialised software, solidThinking Inspire [37]. The optimised models of the connecting elements were also produced from ABS on a 3D printer model, uPrint SE.

Krzyzaniak and Smardzewski [12,34] developed two types of connecting elements that are invisible from the face surface of the joined structural members. The prototypes were obtained by the AM method using a 3D printer, and the elements themselves were made of polyamide DuraForm^®^ ProX^®^ PA Plastic (3D Systems, Rock Hill, SC, USA). The technology by which the individual parts are made is SLS, and the printer is EOS P396 (EOS GmbH, München, Germany). Another team of Polish scientists are also developing connecting elements for furniture, the prototypes of which were created on a 3D printer [13].

Some scientists created a model of auxetic dowels using 3D printing [36,38]. A theoretical and experimental study on the strength of the proposed new solution was carried out. PA12 was used as a material to produce the auxetic dowels, and SLS 3D printing technology was used. A similar development, but for auxetic nails, was developed by Ren et al. [39] and Yao et al. [40], who developed auxetic screws that were fabricated by Selective Laser Melting (SLM) technology. The 3D-printed models were fabricated with a 100 μm nozzle preheated to 200 °C and a raw material particle thickness of 50 μm, and the material was Ti6Al4V (Arcam AB, Gothenburg, Sweden) and had a density of 4.5 g/cm^3^. The printing direction is parallel to the direction of the layers.

Another research study was conducted to investigate and compare the existing material and FDM fabrication method for the designed modular furniture joint [7]. FEM software and a three-point bending test were used to evaluate the structural analysis of the designed modular furniture joint. It was established that the joint withstands a load of up to 730 kg and only weighs 113.59 g. In this research, the materials focused on were ABS and Polyethylene Terephthalate Glycol (PETG).

Aydin [41] designed four types of connectors to assemble a chair instead of a joint used in DIY furniture. CATIA software, v5 R17 was used for the three-dimensional modelling and assembly. Dowels were added to each joint to strengthen the chair structure. The structural elements provided for 3D printing were designed without curves to offer easy-to-manufacture chairs to end users with limited knowledge of wood joints. At the base of the structural connectors is a hollow rectangular parallelepiped with cross-sectional dimensions of 29 mm × 29 mm, a wall thickness of 2 mm, and a length of 31 mm. The materials that can be used are ABS and polylactic acid (PLA).

From the overview of the application of 3D printing, it is clear that this technology opens new opportunities for science and industry, including the furniture sector. Various new design solutions can be experimented with, prototyped, and tested. A small and limited series of design products can be launched where it is not cost-effective to implement industrial technology to produce connecting elements.

### 1.2. Studies to Establish the Strength and Deformation Characteristics of 3D-Printed Joints

Developments in creating and prototyping new joints for furniture using 3D printing are still relatively limited. Polish scientists are pioneers in this field [8,9,11,12,13].

A theoretical model and experimental study analysed the new connectors created by Smardzewski et al. [11]. The 18 mm thick particleboard and MDF test specimens were used. The experimental investigations show that the bending moment and stiffness are more minor when the load is with arm compression. The joints of MDF structural elements have higher bending moment and stiffness ratio values than particleboard.

Another team of Polish scientists created two new models of connecting elements for non-detachable furniture joints produced by 3D printing [12]. An 18 mm laminated particleboard and MDF were used. The loading was accomplished with arm compression and arm opening. The two new connectors exhibit high strengths, 19.55–19.73 N·m and 7.13–7.40 N·m for particleboard and MDF. In the same publication, a theoretical study was carried out to predict the bending moment, and the results were compared with those obtained experimentally.

Podskarbi and Smardzewski [13] developed three new connectors for furniture prototyped on a 3D printer that allows the disassembly of the structure. The authors created a theoretical FEM model to establish the strength and stiffness of the joints and compared it with experimentally obtained results. The structural members are made of a 45 mm thick six-layer HDF composite. The bending moments with arm compression load were between 44 and 54 N·m and between 41 and 50 N·m for arm opening loading. The stiffness coefficients were between 800 and 1700 N·m/rad for arm compression loading and between 920 and 1340 N·m/rad for arm opening loading.

Another study on the strength characteristics of 3D-printed connecting elements was carried out by Nikolau et al. [42]. Using a 3D printer and PLA as a material, they designed and fabricated corner joints from larch (*Larix decidua* Mill.) solid wood elements. Microscopic studies analysed different printing defects and their effect on the strength characteristics. The results are compared with the most popular joint for solid wood elements, the mortise and tenon joint. The authors conclude that lay-up direction correlates with the joints’ strength. The bending moment with arm compression load of the joints using 3D-printed connecting elements was about 58% lower than that of the joints using mortise and tenon.

Other researchers created two joining elements made by the 3D printer, designed to join elements of solid spruce wood (*Picea abies* Karst.) lengthwise [43]. An Ultimaker 3 3D printer (Ultimaker B.V., Geldermalsen, The Netherlands) and PLA filament were used. The wall thickness of the connecting elements was 2 mm. The joints were made by gluing them with one-part polyurethane adhesive (Kleiberit 501, Kleiberit Se & Co. Kg Weingarten, Germany). A theoretical FEM analysis of the stresses and strains was performed, and it was found that the strength and strain of the 3D-printed joined parts were significantly lower than those of the solid spruce wood beam. For both models of connectors, the theoretically calculated deflections did not match the deflection obtained in testing.

Three different surface designs of the dowels, including grooved, straight, and spiral, made by the 3D printer, produced by using PLA and PLA+ Thermoplastic Polyurethane (TPU) and resin (Acrylic Photopolymer), were investigated and compared to wood and plastic dowels [44]. The test samples were subjected to a shear test. The results of this study demonstrated the potential of 3D printing technology in furniture manufacturing with different designs, such as the surface design for the dowel.

Based on the study, it can be concluded that 3D printing technology is increasingly entering various manufacturing areas, including furniture. No 3D-printed connecting elements for thin structural elements were found in the literature, nor were studies on their strength and deformation characteristics. For the present study, connecting elements have been developed by 3D printing and designed to join thin structural elements. A new joining element has also been created where there is no gap between the panels. Ten types of joints made with plywood and MDF panels were investigated, and their strength and deformation characteristics under the arm compression test were determined, as no data on the strength characteristics of such connectors were found. All joints exhibit very high bending strength with an arm compression test and relatively good stiffness. Furniture designers and manufacturers can use such connectors without worrying about their performance, as proved by the high strength and stiffness of the results obtained.

## 2. Materials and Methods

Ten types of joints with 3D-printed connecting elements were selected for this study. End corner joints for thin structural elements with 6 mm, 9 mm, and 12 mm thicknesses were developed. The first two structural elements fall into the category of ultra-thin panels (under 10 mm) and the third into the group of thin panels (10–15 mm) [45]. One corner joint requires two connecting elements made by 3D printing technology. For the study, 120 connecting elements were designed and fabricated, and 60 joints were tested. Each type of joint has at least five identical samples.

### 2.1. Material and Technology for Manufacturing 3D-Printed Connecting Elements

A 3D professional printer manufactured by 3Dgence, model DOUBLE P255, produced the 3D-printed connecting elements. The 3D models of connecting elements created on 3DSmax 2020 software are imported into 3DGense Slicer 4.0 software. This program creates a print file and controls the number of solid layers in all directions and the type and percentage of hollow section fill.

Material selection is of utmost importance for the successful creation of any product, including those obtained through AM. In AM, the following materials are used: PLA, PETG, and ABS. PLA is a biopolymer created from plant products (corn), has excellent physical and mechanical properties, and is, therefore, widely used as a material in FDM technology. The use of PLA-based bio-composites/green composites is progressively increasing. Due to environmental concerns, PLA-based bio-composites are among the best alternatives to replace non-biodegradable petroleum-derived products worldwide. These composites offer acceptable mechanical and thermal properties, making them suitable replacements for existing petroleum-based, non-biodegradable materials [46].

Due to the advantages mentioned above, a biodegradable thermoplastic polymer, PLA filament, was used in this study to produce the connecting elements. Material jetting was used to create objects similar to a two-dimensional inkjet printer. The material is applied to a build platform using a continuous or ‘Drop on Demand’ (DOD) approach.

Parameters of the 3D printer and selected printing mode:

- Temperature of ceramic plate (bed)—65 °C
- XY positioning accuracy—6 µ
- Z positioning accuracy—0.4 µ
- Material used—PLA (Polylactic Acid)
- Diameter of material used—1.75 mm
- Nozzle temperature—210 °C
- Nozzle diameter—0.4 mm
- Layer height—0.25 mm
- Speed of filling material application—70 mm/s
- Printing speed of lower and upper layers—45 mm/s
- Printing speed of outer part walls—30 mm/s
- Printing speed of inner walls—40 mm/s
- Cavity filling—20%

### 2.2. Design Concept of the Joints for Thin and Ultrathin Structural Elements Made by 3D Printing

**Figure 1:**
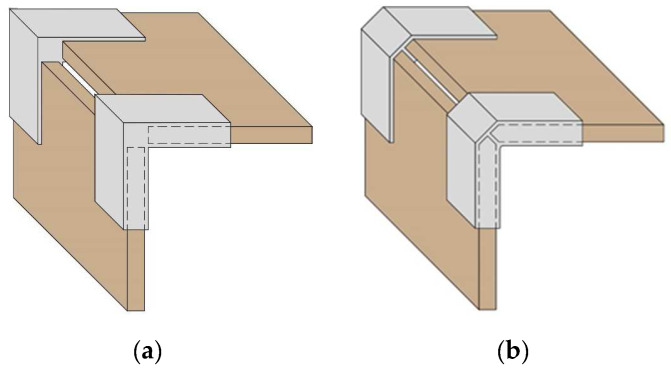
General view of detachable end corner joints of thin structural elements by 3D-printed connecting elements: (**a**) without chamfer in the edge; (**b**) with chamfer in the edge.

For this study, two variations of end corner joints were selected, with and without chamfering along the edge of the structural elements to be joined (Figure 1). The variant of the joint without chamfering along the edge of the joined structural elements is similar to existing specimens in the literature (Figure 1a). The edge-chamfered connecting element is newly developed and patented by the authors. Its main advantage is that it eliminates the spacing/gap between the structural elements to be joined (Figure 1b).

The joints are made of panel-based materials, plywood (9 and 12 mm) and MDF (6 mm). Both materials have excellent mechanical characteristics and are suitable for thin and ultra-thin plate structures [10,47,48,49].

### 2.3. Type and Dimensions of the Joints

**Figure 2:**
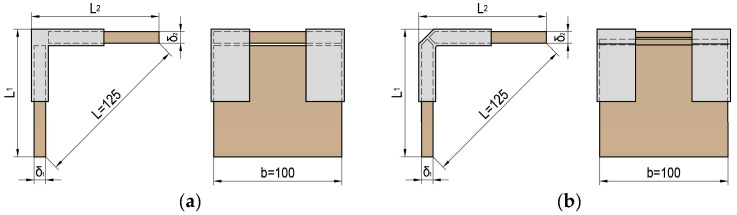
Type and dimensions of test samples of end corner joints of panel structural elements: (**a**) without chamfer in the edge; (**b**) with chamfer in the edge.

The type and dimensions of the joints are according to the methodology for testing panel structural elements given by Kyuchukov and Jivkov [50] and are shown in Figure 2. The distance L between the inner edges of the test samples is 125 mm. The lengths of the test samples L_1_ and L_2_ are equal.

**Figure 3:**
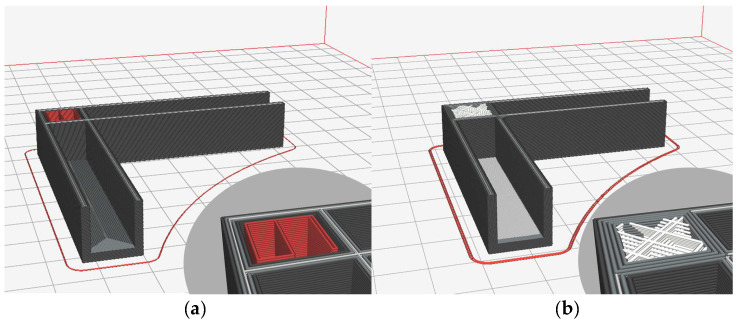
A 3D-printed connecting element: (**a**) with zig-zag filling of hollow section in red colour; (**b**) with cross-filling of hollow section in white colour and internal radius of 2 mm.

Figure 3 shows the 3D-printed connecting elements, implemented in two variations of filling the hollow section between its arms and two wall thicknesses, 1.5 and 2 mm. Figure 3a shows a zig-zag filling of the cavity; in Figure 3b, cross-filling is shown. In the two versions, besides the different filling of the cavity, the arrangement of the layers at the base of the connecting elements is also different.

**Figure 4:**
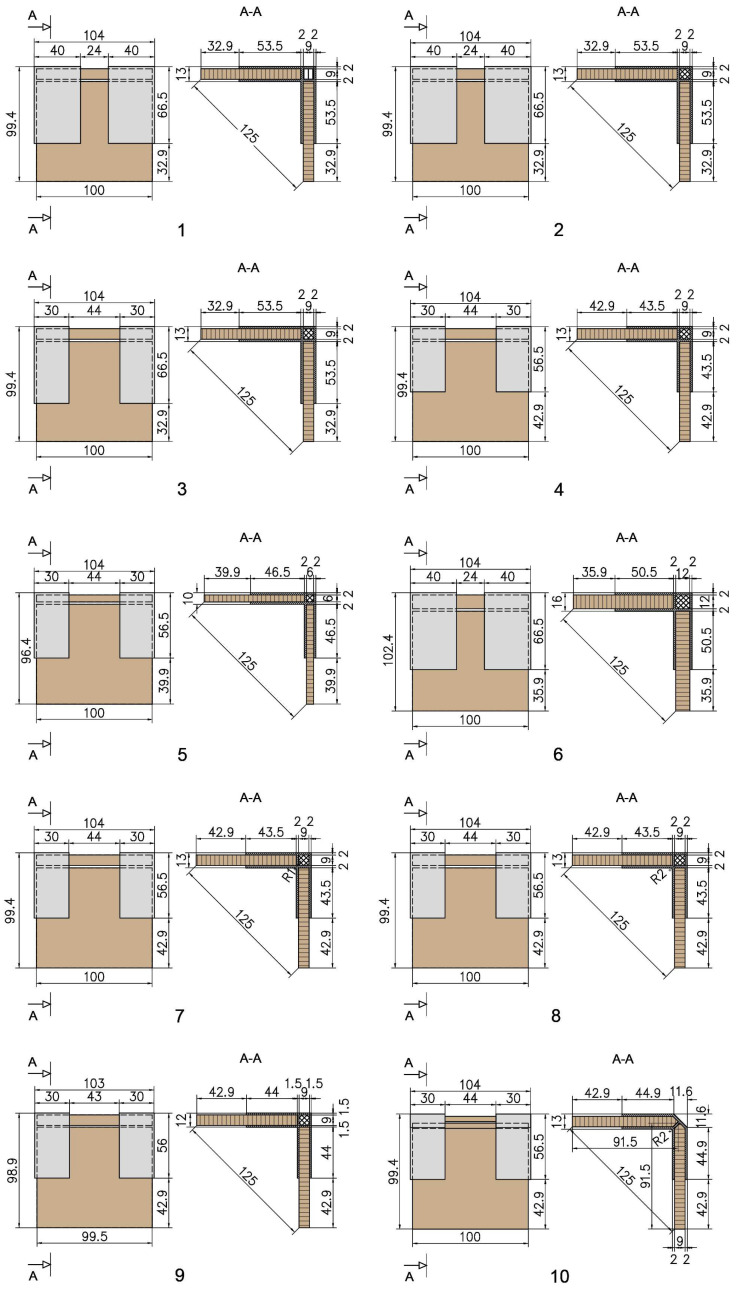
Detachable end corner joints of thin and ultra-thin structural elements by 3D-printed connecting elements: (**1**) 9 mm plywood joint with connecting element 66.5 × 66.5 × 40 mm and zig-zag filling of the hollow section and sequential printing of the layers in the base (1_Ply_9_X120×40); (**2**) 9 mm plywood joint with connecting element 66.5 × 66.5 × 40 mm, cross-filling of the hollow section and combined printing of the layers at the base of the connecting element (2_Ply_9_X120×40C); (**3**) 9 mm plywood joint with connecting element 66.5 × 66.5 × 30 mm, cross-filling of the hollow section and combined printing of the layers at the base of the connecting element (3_Ply_9_X120×30C); (**4**) 9 mm plywood joint with connecting element 56.5 × 56.5 × 30 mm, cross-filling of the hollow section and combined printing of the layers at the base of the connecting element (4_Ply_9_X100×30C); (**5**) 6 mm MDF joint with connecting element 56.5 × 56.5 × 30 mm, cross-filling of the hollow section and combined printing of the layers at the base of the connecting element (5_MDF_6_X100×30C); (**6**) 12 mm plywood joint with connecting element 66.5 × 66.5 × 40 mm, cross-filling of the hollow section and combined printing of the layers at the base of the connecting element (6_Ply_12_X120×40C); (**7**) 9 mm plywood joint with connecting element 56.5 × 56.5 × 30 mm, cross-filling of the hollow section and combined printing of the layers at the base of the connecting element and radius R1 between the arms of the connecting element (7_Ply_9_X100×30C_R1); (**8**) 9 mm plywood joint with connecting element 56.5 × 56.5 × 30 mm, cross-filling of the hollow section and combined printing of the layers at the base of the connecting element and radius R1 between the arms of the connecting element (8_Ply_9_X100×30C_R2); (**9**) 9 mm plywood joint with connecting element 56 × 56 × 30 mm, cross-filling of the hollow section and combined printing of the layers at the base of the connecting element and 1.5 mm wall thickness of the connecting element (9_Ply_9_X100×30C_δ1,5); (**10**) 9 mm plywood joint with connecting element 56.5 × 56.5 × 30 mm and internal radius of 2 mm and double-sided bevel at the panel edge (10_Ply_9_+X100×30_R2).

The type and parameters of all joints made by 3D-printed connecting elements are shown in Figure 4 and described in Table 1.

### 2.4. Test Methods

Joint strength and stiffness are often established by testing them under bending, tensile, and shear loading. It is accepted that the most critical loads for joints in furniture construction are bending loads [50]. Therefore, the selected criteria for evaluating the strength and deformation characteristics of the joints of the present work are the bending moment and the stiffness under bending load, respectively. All the joints in this work were tested under bending load with arm compression. The reason for this choice is the results obtained from many other studies, which found that bending moments and stiffnesses are often lower than those under the arm opening test [11,12,13,51,52,53,54,55,56,57,58]. This makes the bending load with arm compression critical for the strength and deformation capacity of the furniture structure.

**Figure 5:**
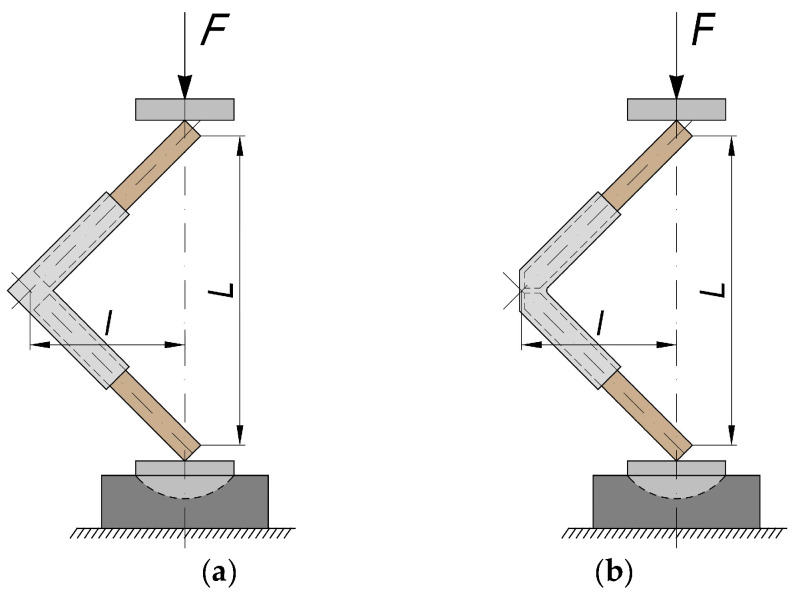
Test scheme for end corner joints of thin structural elements under bending load with arm compression and determination of the bending arm: (**a**) end corner joint, (**b**) mitre joint.

**Figure 6:**
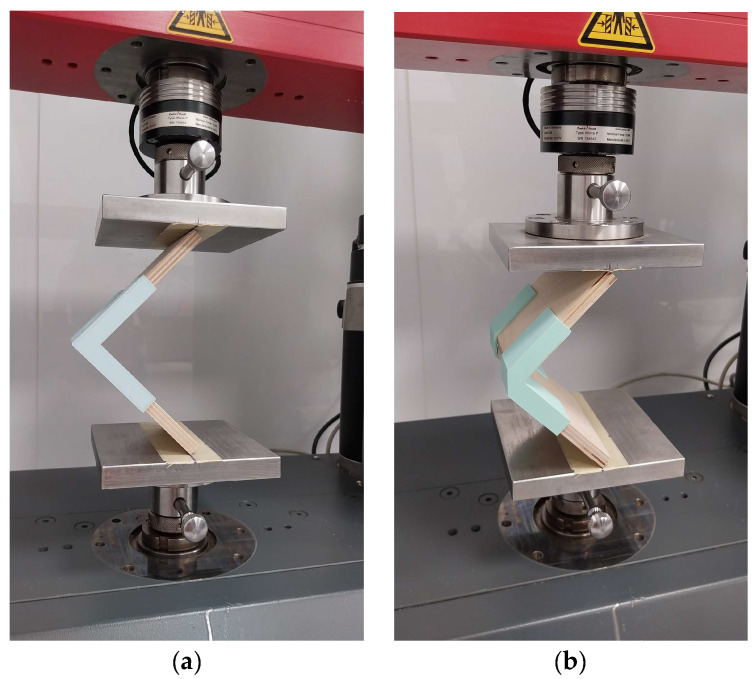
Test samples of thin structural elements under bending load with arm compression during testing: (**a**) end corner joint, (**b**) mitre joint.

Figure 5 shows the general scheme of the bending load test with arm compression and how the arm (l) is determined, where F is the loading force and L is the distance between the arms. The loading speed was adjusted so that the time to rupture is within 60 ± 30 s. Figure 6 shows the test samples during testing on a universal testing machine.

The criterion for determining the strength of the tested joints is the maximum bending moment, M_max_, calculated according to the following formula:

M_max_ = F · l, (1)

where F is the maximum force under arm compression bending in Newtons, and l is the ar in, meter.

**Figure 7:**
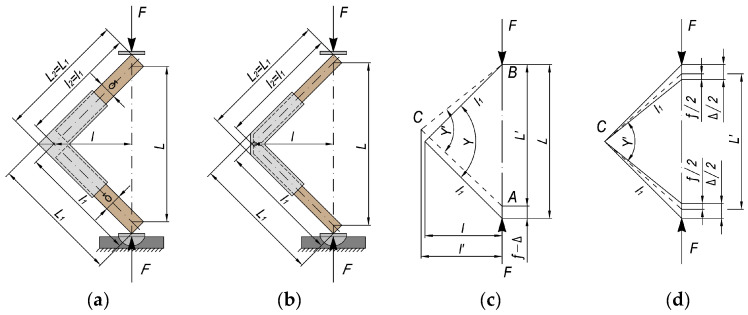
Test scheme and deformation under arm compression bending load of the test samples: (**a**) type of loading and dimensions of the tested samples of end corner joints; (**b**) type of loading and dimensions of the tested samples of mitre corner joints; (**c**) scheme of loading and change in arm length; (**d**) determination of the deformation of the tested samples.

The stiffness coefficient, c, is the criterion for determining the deformation characteristics of the corner joints. A methodology by Kyuchukov and Jivkov [50] was used to determine the deformation characteristics of the joints, in which the strength and deformation characteristics are simultaneously established. The type of test samples and the bending load scheme are given in Figure 7.

When the joints are loaded in bending under arm compression, a deformation occurs in which the right angle between the arms of the joint begins to decrease, and the bending arm, l, increases. The linear displacement, *f_i_*, of the application points of the forces, *F_i_*, is recorded for each test sample at the level of 10 and 40% of the maximum load. It represents a sum of displacement resulting from turning the joint arms and additional displacement, Δ*_i_*, resulting from bending of the arms.

The displacement, Δ*i,* is calculated by the following formula:

(2) $$\Delta_{i}=\frac{F_{i}l^{3}}{3EI},$$

where *F_i_* is the magnitude of the load forces with arm compression, N; *a* is the axial length of the joint arms, m; *E* is the modulus of elasticity, N/mm^2^; and *I* is the axial moment of inertia of the cross-section of the joint arms, m^4^, which is calculated by the following formula:

(3) $$I=\frac{\delta b^{3}}{12},$$

where *b* is the width of the arms, m, and *δ* is the thickness of the arms, m.

The formula determines the distance between the force application points at each level of loading:

(4) $$L_{1}=L-f_{i}+\Delta_{i},$$

The angle $\gamma_{i}$ (rad) changed under loading between the joint arms is calculated by the following formula:

(5) $$\gamma_{i}=2\arcsin\frac{L_{i}}{2a}=2\arcsin\frac{L-f_{i}+\Delta_{i}}{2a},$$

The changed bending arm, *l_i_*, is determined by the following formula:

(6) $$l_{i}=a\cos\frac{\gamma_{i}}{2},$$

The result from the deformation under the compression bending test is the semi-rigid rotation of the joint arms in radians (rad).

(7) $$\alpha_{i}=\frac{\pi }{2}-\gamma_{i},$$

For 10 and 40% of the load force, *F_i_*, the bending moment in N·m is calculated according to the following formula:

(8) $$M_{i}=F_{i}l_{i},$$

The stiffness coefficient under the compression bending test, *c_i_* (N·m/rad), is calculated by the following formula:

(9) $$c_{i}=\frac{\Delta M_{i}}{\Delta \alpha_{i}},$$

In (8), the following designations are used:

(10) $$\Delta M_{i}=M_{i}-M_{0}$$

(11) $$\Delta \alpha_{i}=\alpha_{i}-\alpha_{0}$$

where *M_i_* and *α_i_* are determined according to (8) and (7), respectively, for the value of force, *F_i_*, equal to 40% of *F_max_*, and *M*_0_ and *α*_0_ are determined according to (8) and (7) or the value of force, *F*_0_, equal to 10% of *F_max_*.

The stiffness coefficient, c, is a deformation characteristic of the corner joint under the compression bending test. It is defined as the arithmetic mean of the result of (9) for each test sample when loaded in the section corresponding to the linear section on the curve of the correlation between the bending moment and the corner deformation of the joint.

The test samples were tested on a Zwick/Roell Z010 universal testing machine (ZwickRoell GmbH & Co. KG, Ulm, Germany) at the University of Chemical Technology and Metallurgy (CCTM) in the Department of Pulp, Paper, and Printing, Sofia, Bulgaria. The tests were carried out at 20 ± 2 °C and a relative humidity of 55 ± 5%.

### 2.5. Statistical Processing

A descriptive statistical analysis of the results was conducted with XLSTAT statistical and data analysis solution Lumivero (2024) [59]. A one-way ANOVA was performed on the results for the bending strength and stiffness coefficient of corner joints to analyse variance at a 95% confidence interval (*p* < 0.05). The statistical differences between mean values were evaluated using Tukey’s honest significant difference (HSD) post hoc test.

## 3. Results

### 3.1. Bending Moments of the Joints

The results for the bending moment of end-corner joints of thin and ultra-thin materials are given in Table 2, which shows Tukey’s HSD analysis of the differences between the groups with a confidence interval of 95% of bending capacity of mitre joints made of thin and ultra-thin structural elements of all pairwise comparisons. The joints are ranked in descending order of bending moments. The results are divided into three groups of homogeneity.

From the results obtained, it can be seen that the joints with the highest bending moment (44.16 N·m) are the joints of structural elements made of 12 mm plywood and connecting elements with dimensions 66.5 mm × 66.5 mm × 40 mm and cross-filling of hollow section (6_Ply_12_X120×40C), and secondly, the structural elements of 9 mm plywood and the same type and size of connecting element (2_Ply_9_X120×40C), 36.88 N·m. Next are the joints of 9 mm-thick plywood structural elements where the width of the joint element is 30 mm and not 40 mm as in the former. The joints of 9 mm-thick plywood structural elements and connecting elements 66.5 mm × 66.5 mm × 40 mm with zig-zag hollow section filling have the lowest bending moment. Similar strength was shown in the joints of 6 mm MDF (5_MDF_6_X100×30C) and 9 mm plywood and connecting elements with dimensions 56.5 mm × 56.5 mm × 30 mm, zig-zag filling of the hollow section (9_Ply_9_X100×30C_δ1,5), and wall thickness δ = 1.5 mm. The difference between the bending moments of the joint with the most extensive bending moment and the one with the smallest is almost double.

**Figure 8:**
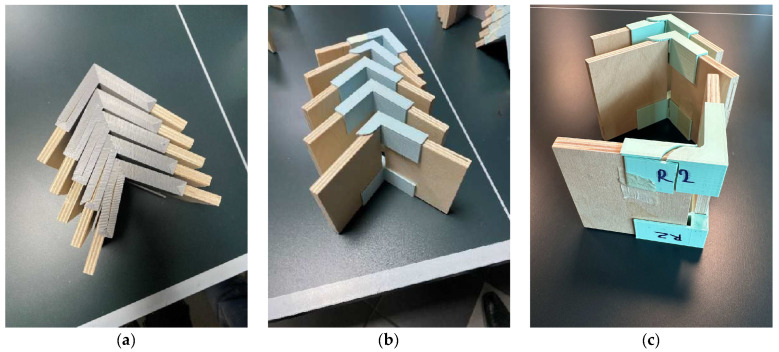

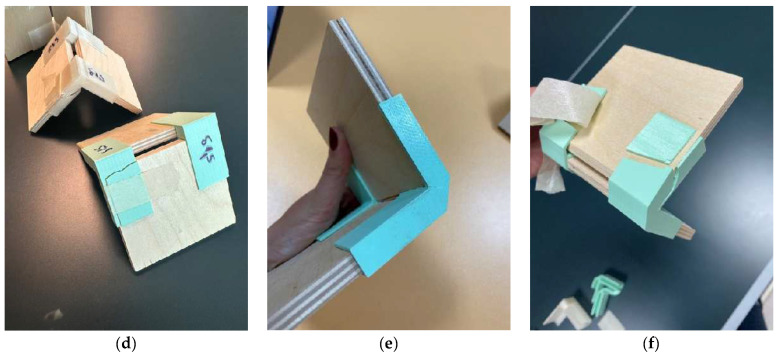
The typical mode of failure of the test samples under arm compression load with 3D-printed connecting elements: (**a**) connecting element 66.5 mm × 66.5 mm × 40 mm and zig-zag filling of the hollow section for 9 mm-thick plywood (1_Ply_9_X120×40) destruction parallel to the layers; (**b**) connecting element 56.5 mm × 56.5 mm × 30 mm and cross-filling of hollow section for structural elements with 9 mm-thick plywood (4_Ply_9_X100×30C)—combined destruction along the layers and perpendicular to the layers; (**c**) connecting element 56.5 mm × 56.5 mm × 30 mm and cross-filling of hollow section and internal radius 2 mm for structural elements with 9 mm-thick plywood—perpendicular destruction (8_Ply_9_X100×30C_R2); (**d**) connecting element 56.5 mm × 56.5 mm × 30 mm and cross-filling of hollow section and wall thickness 1.5 mm for structural elements with 9 mm-thick plywood (9_Ply_9_X100×30C_δ1,5); (**e**,**f**) connecting element 56.5 mm × 56.5 mm × 30 mm and internal radius 2 mm for structural elements with 9 mm-thick plywood and double-sided bevel at the edge (10_Ply_9_+X100×30_R2).

The typical failure mode of all the joints happens in the area of 3D-printed connecting elements. Failure occurs both along the ply line of the joints and across the plies (Figure 8). It is most commonly found in the inner corner between the shoulders of the joint element. In the case of joints using 3D-printed 66.5 mm × 66.5 mm × 40 mm connecting elements and zig-zag filling of the hollow section of 9 mm-thick plywood structural members, the failure is frontal along the layers of the connecting element (Figure 8a). The typical failure at this joint is due to the orientation of the plies at the base of the connecting element, where they run sequentially. The failure in all other joints, from 2 to 10, is combined along the layers and perpendicular to the layers of the connecting elements. In these joints, the orientation of the layers at the base of the connecting elements is combined, with the first five layers running consecutively around the perimeter and the subsequent layers oriented crosswise at 45°. Figure 8b–f shows some of these failure modes.

### 3.2. Stiffness of the Joints

Table 3 gives the data for the stiffness coefficients according to the Tukey HSD test for a significant difference of α = 0.05 at the 95% confidence level. Four homogeneity groups were found, with the highest stiffness coefficient (348.12 N·m/rad) being the joints of 12 mm-thick plywood and 66.5 mm × 66.5 mm × 40 mm cross-filled hollow section connecting elements (6_Ply_12_X120×40C). This is also the joint with the most significant bending moment. Also, the joints of plywood structural elements with a thickness of 9 mm joined with connecting elements with lengths L = 56.5 and L = 66.5 mm and widths B = 30 and B = 40 mm are characterised by high stiffness. The 9 mm-thick plywood joints and 56.5 mm × 56.5 mm × 30 mm joints with δ = 1.5 mm wall thickness and cross-filling of hollow section (9_Ply_9_X100×30C_δ1,5) have the lowest stiffness coefficient. With almost the same stiffness coefficients are the joints made of 6 mm-thick MDF (5_MDF_6_X100×30C). From the results obtained, it cannot be proved that increasing the radius in the angle between the arms from R = 1 to R = 2 mm also increases the stiffness.

## 4. Discussion

The joints investigated in this study by 3D-printed connecting elements generally show high bending strength when loaded with arm compression. No studies of similar joints in the literature can be used for comparison. However, a comparison could be made with other conventional joints. For example, the joint of structural elements made of 12 mm-thick plywood and connecting elements with dimensions 66.5 mm × 66.5 mm × 40 mm and cross-filling of hollow section (6_Ply_12_X120×40C), with a bending moment of 44.16 N·m, has about 16% higher strength than mitre joints by gluing of 12 mm-thick plywood, which has a bending moment of 37.97 N·m and is five times bigger than a joint with Lamello 0, with a bending moment of 9.07 N·m [60]. Compared to the detachable mitre joint by the eccentric connector of 12 mm-thick plywood and MDF, the joint with the 3D-printed connecting element (6_Ply_12_X120×40C) showed 28–29% higher strength [60]. The results obtained for the bending moments in this study for all types of joints are higher than the results obtained by Krizanyak and Smardzewski [12], who also proposed joints manufactured from polyamide DuraForm^®^ ProX^®^ PA Plastic (3D Systems, Rock Hill, SC, USA)using 3D printing. The joints were constructed with two connectors; the material was particleboard and MDF, with a thickness of 18 mm, and a bending moment was obtained from 7.13 to 19.75 N·m.

From the results, it can be suggested that the colour of the filament probably also influences the strength of the compound. This may be the subject of future research. Another problem noticed during the fabrication of the connecting elements is the sensitivity of the process from the nozzle temperature setting. A small change in the operating temperature in either direction can lead to defects in the printed part due to the specificity of different PLA filament colours to temperature. This can be explained by the different characteristics of the fillers used to acquire the respective colour. Other researchers have identified a similar problem [28].

Unlike bending moments, the stiffness coefficients of 3D-printed joints are not as high as conventional glued joints. For example, the joint of structural elements made of 12 mm-thick plywood and connecting elements, with dimensions 66.5 mm × 66.5 mm × 40 mm and cross-filling of hollow section (6_Ply_12_X120×40C), with a stiffness coefficient of 348 N·m/rad, has almost a three and a half times lower stiffness than mitre joints by gluing of 12 mm-thick plywood, which has a stiffness coefficient of 1220 N·m/rad [60]. Still, the joints with Lamello have close to this stiffness at 299 N·m/rad [60]. The situation is reversed when comparing joints with 3D-printed connecting elements with detachable conventional mitre joints. Compared to the detachable mitre joint by the eccentric connector of 12 mm-thick plywood and MDF, the joint with 3D-printed connecting elements showed three to five times higher stiffness [60]. On the other hand, the connectors proposed by Krizanyak and Smardzewski [12] have about five times higher stiffness coefficients. However, this has its explanation. Firstly, they are for joints with a thickness of 18 mm. Secondly, the connectors are embedded in the panel, in contrast to the proposal in this study for 3D-printed connecting elements.

One possibility that could be worked on in the future is to add some light ribbing along the arms of the connecting elements. The ribbing adds stiffness and strength to the whole product’s structure without increasing the thickness of the walls [61]. Thus, the joint itself will have a high flexural strength and stiffness coefficient. Another alternative option is to look into adding a locking pin.

## 5. Conclusions

This study provides a new perspective on using 3D printing in furniture construction. In this way, newly developed connectors can be prototyped quickly and cheaply, or they can be produced for small series or boutique furniture. The excellent bending strength and good stiffness of the joints made of the thin and ultra-thin panels in this study are a testament to their applicability. By using 3D technology, some design disadvantages can be cleared, and production can proceed by traditional injection moulding methods. Studies of comparable joints obtained by 3D printing were not found in the literature, and therefore, the results were compared only with conventional joints.

From the research carried out to establish the bending moments and stiffness coefficients of detachable end-corner joints of thin and ultra-thin structural elements using 3D-printed connecting elements, the following more essential conclusions can be drawn:

- All 3D-printed connecting elements give the joints a very high bending strength when loaded in arm compression.
- The stiffness coefficients of joints with 3D-printed connecting elements are higher than those of conventional detachable mitre joints but lower than those of glued ones.
- The difference in the bending moment of the joints of 9 mm- and 12 mm-thick plywood with the exact parameters of the 3D-printed connecting elements was 19.7%, and in the stiffness coefficients it was 11.95%.
- The cross-filling of the hollow section of the connecting elements increases the joints’ strength and stiffness.
- Reducing the width of the connecting elements from 40 mm to 30 mm does not significantly affect the joints’ strength and stiffness coefficients.
- Reducing the wall thickness of the connecting elements from 2 to 1.5 mm reduces strength by almost 32% and stiffness coefficients by 42%.
- No significant difference was found in the strength and stiffness coefficients of joints where the inner radius between the arms of the connecting element was 1 or 2 mm.

## 6. Patents

Due to the present research, a utility model has been registered, Petrova, B., Jivkov, V. Certificate for “Utility model” registration №4316 U1, Application №5486/10.03.2022. Multifunctional connecting element for standard and thin panel structural elements, doors and backs in furniture. Published in PV Bulletin No. 202209, 15 September 2022.

## Figures and Tables

**Table 1:** Material type, parameters, and index of the joints made by 3D-printed connecting elements.

№	Material Type	Thickness, mm	Joint Type	Filling of Hollow Section	Dimensions of the Connecting Element, mm	Index of the Joints
1	Plywood	9	without chamfer	zig-zag	66.5 × 66.5 × 40	1_Ply_9_X120×40
2	Plywood	9	without chamfer	cross	66.5 × 66.5 × 40	2_Ply_9_X120×40C
3	Plywood	9	without chamfer without chamfer	cross	66.5 × 66.5 × 30	3_Ply_9_X120×30C
4	Plywood	9		cross	56.5 × 56.5 × 30	4_Ply_9_X100×30C
5	MDF	6	without chamfer without chamfer	cross	56.5 × 56.5 × 30	5_MDF_6_X100×30C
6	Plywood	12		cross	66.5 × 66.5 × 40	6_Ply_12_X120×40C
7	Plywood	9	without chamfer without chamfer	cross	56.5 × 56.5 × 30	7_Ply_9_X100×30C_R1
8	Plywood	9		cross	56.5 × 56.5 × 30	8_Ply_9_X100×30C_R2
9	Plywood	9	without chamfer with chamfer	cross	56.5 × 56.5 × 30	9_Ply_9_X100×30C_δ1,5
10	Plywood	9		-	56.5 × 56.5 × 30	10_Ply_9_+X100×30_R2

**Table 2:** Tukey’s HSD analysis results of the differences between the groups with a confidence interval of 95% on the bending moments of end corner joints made of thin and ultra-thin structural elements by 3D-printed connecting elements.

No	Index of the Joints	M, N·m	Standard Error, N·m	Homogeneity Groups		
				1	2	3
1	6_Ply_12_X120×40C	44.16	1.51	A		
2	2_Ply_9_X120×40C	36.88	1.79	A	B	
3	3_Ply_9_X120×30C	35.88	2.31		B	
4	8_Ply_9_X100×30C_R2	34.01	1.51		B	
5	7_Ply_9_X100×30C_R1	33.94	1.79		B	
6	4_Ply_9_X100×30C	33.27	1.51		B	
7	10_Ply_9_+X100×30_R2	28.90	1.51		B	C
8	9_Ply_9_X100×30C_δ1,5	24.53	2.31			C
9	5_MDF_6_X100×30C	24.24	1.63			C
10	1_Ply_9_X120×40	24.02	1.79			C

**Table 3:** Tukey’s HSD analysis results of the differences between the groups with a confidence interval of 95% on the stiffness coefficients of end corner joints made of thin and ultra-thin structural elements by 3D-printed connecting elements.

No	Index of the Joints	c, N·m/rad	Standard Error, N·m/rad	Homogeneity Groups			
				**1**	**2**	**3**	**4**
1	6_Ply_12_X120×40C	348.12	1.77	A			
2	2_Ply_9_X120×40C	310.98	1.77	A	B		
3	3_Ply_9_X120×30C	289.20	1.77	A	B		
4	4_Ply_9_X100×30C	250.19	1.98		B	C	
5	7_Ply_9_X100×30C_R1	249.05	1.77		B	C	
6	8_Ply_9_X100×30C_R2	241.61	1.62		B	C	
7	10_Ply_9_+X100×30_R2	178.23	1.77			C	D
8	1_Ply_9_X120×40	164.48	1.77			C	D
9	5_MDF_6_X100×30C	147.07	2.80			C	D
10	9_Ply_9_X100×30C_δ1,5	145.32	1.77				D

## Data Availability

Data are contained within the article.
